# The Devastating Health Consequences of the Ohio Derailment: A Closer Look at the Effects of Vinyl Chloride Spill

**DOI:** 10.3390/ijerph20065032

**Published:** 2023-03-13

**Authors:** Wenjie Sun

**Affiliations:** Center for Health Sciences, Oklahoma State University, 1111 W 17th Street, Tulsa, OK 74107, USA; wenjie.sun@okstate.edu

On 3 February 2023, a train derailed in the village of East Palestine, Ohio, home to approximately 4700 residents. Approximately 1.1 million pounds of vinyl chloride, a toxic flammable gas, was released and quickly became volatile [1]. Vinyl chloride exposure is associated with an increased risk of rare forms of liver cancer, such as hepatic angiosarcoma [2], as well as primary liver cancer (hepatocellular carcinoma) [3], brain cancer [4], and lung cancer [5]. The accident caused an extremely large volume of burning gas to be released, which could generate dioxins and derivatives, notorious carcinogens that can exist in air, soil, and water for many years.

The inhalation of certain irritant gases, such as chlorine, at sufficiently high acute doses can cause bronchiolitis obliterans, a rare disease involving concentric bronchiolar fibrosis in a few months [6]. Small airway fibrosis is one of the pathological characteristics of COPD. Although there is no solid evidence to show that the vinyl chloride spill directly results in COPD, studies addressing whether individuals, especially children, who were exposed to vinyl chloride are at a heightened risk for COPD are warranted. Those with the restrictive-only pattern had evidence of true lung restriction and were at increased risk of multimorbidity by middle age, whereas people can benefit from early COPD interventions [7].

Moreover, toxic chemical disasters pose a high risk of psychological trauma for nearby residents. A wide spectrum of psychological distress, including the acute onset of psychiatric disorders and the exacerbation of existing psychological and psychiatric conditions, has been seen at accident sites [8]. Hence, hospital management should include early involvement of mental health services. Long-term effects, such as PTSD and suicide, should also be monitored in the community. Additionally, a significant increase in hypertension-related hospital discharge rates was observed in the years following the chlorine spill from a train derailment with toxic chemical leakage [9].

Therefore, health assessments should include a comprehensive evaluation of the residents’ medical records, as well as the collection of data on any health effects experienced by the surrounding community regarding cancer, COPD, cardiovascular diseases, mental health, etc. Third-party scientific opinions are warranted to calm down the panic among the community and should acknowledge uncertainty and inform the public about what is known and unknown [10]. Any miscommunication or hidden potential consequences may lead to distrust and a secondary disaster, as shown in Chernobyl and Fukushima.

## References

[1] Hauser C. (2023). After the Ohio Train Derailment: Evacuations, Toxic Chemicals and Water Worries. https://www.nytimes.com/article/ohio-train-derailment.html?action=click&module=Well&pgtype=Homepage&section=US%20News.

[2] Zheng Y.W., Zhang X.W., Zhang J.L., Hui Z.Z., Du W.J., Li R.M., Ren X.B. (2014). Primary hepatic angiosarcoma and potential treatment options. J. Gastroenterol. Hepatol..

[3] Mundt K.A., Dell L.D., Crawford L., Gallagher A.E. (2017). Quantitative estimated exposure to vinyl chloride and risk of angiosarcoma of the liver and hepatocellular cancer in the US industry-wide vinyl chloride cohort: Mortality update through 2013. Occup. Environ. Med..

[4] Rodrigues E.G., Herrick R.F., Stewart J., Palacios H., Laden F., Clark W., Delzell E. (2020). Case-control study of brain and other central nervous system cancer among workers at semiconductor and storage device manufacturing facilities. Occup. Environ. Med..

[5] Girardi P., Barbiero F., Baccini M., Comba P., Pirastu R., Mastrangelo G., Ballarin M.N., Biggeri A., Fedeli U. (2022). Mortality for Lung Cancer among PVC Baggers Employed in the Vinyl Chloride Industry. Int. J. Environ. Res. Public Health.

[6] Kerger B.D., Fedoruk M.J. (2015). Pathology, toxicology, and latency of irritant gases known to cause bronchiolitis obliterans disease: Does diacetyl fit the pattern?. Toxicol. Rep..

[7] Dharmage S.C., Bui D.S., Lowe A.J., Thompson B., Bowatte G., Thomas P., Garcia-Aymerich J., Jarvis D., Hamilton G.S., Johns D.P. (2022). Thompson Lifetime spirometry patterns of obstruction and restriction, and their risk factors and outcomes: A prospective cohort study. Lancet Respir. Med..

[8] MCCormick L.C., Tajeu G.S., Klapow J. (2015). Mental health consequences of chemical and radiologic emergencies: A systematic review. Emerg. Med. Clin. N. Am..

[9] Howell A.V., Vena J.E., Cai B., Lackland D.T., Ingram L.A., Lawson A.B., Svendsen E.R. (2019). Temporal Trends in Cardiovascular Hospital Discharges Following a Mass Chlorine Exposure Event in Graniteville, South Carolina. Front. Public Health.

[10] Piltch-Loeb R., Savoia E., Wright N., Gupta R. (2018). Facilitated Look-Back Analysis of Public Health Emergencies to Enhance Preparedness: A Brief Report of a Chemical Spill in Charleston, West Virginia. J. Public Health Manag. Pract..

